# False Teeth Swallowing

**Published:** 1858-07

**Authors:** 


### ARTICLE XVI.

### *False Teeth Swallowing.*

MESSRS. EDITORS,—The Journal has lately had a number of cases of false teeth swallowing. Will you accept the following one, in which such teeth were not swallowed exactly, but produced trouble both to the patient and his physicians.

I was once called to visit a neighbor, a well informed and ingenious mechanic, who had fallen from a height, and was thought to be seriously injured. Upon reaching the address,

I found the late Mr. J. C., stretched out on the floor of his shop, in apparently an unconscious state, breathing very stertorously. He had, just before, fallen from a ladder of common height, and had not moved or spoken since. Examination discovered no hurt to head or limb. There was some contusion about the chin and mouth. Surgery not being one of my specialties, and I not reaching a satisfactory diagnosis, sent for my friend, the late Dr.——,* quite distinguished among our many very skillful surgeons. He looked Mr. — very carefully over, as the phrase is in the Old Colony (at least I never heard it used any where else, and my travels at home and abroad have not been small) and could find no cause for the present state of things. The pulse indicated neither concussion nor compression. There was no vomiting. The temperature was good. So having made arrangements for applications to the head and feet, hoping to strike the morbid condition, or its cause, as it probably lay somewhere between these two extremes of the body lying senseless before us, and as remedies were necessarily confined to the surface, swallowing being impossible—having, in short, done what we could, we left agreeing to meet at the same place in the afternoon.

We met as agreed upon, and to our surprise and joy found Mr. J. C. up, and as bright and joyous as I ever had seen him. "I am well again you see," said he. You could not find out my disease or its cause. I will tell you; my ladder fell, and I fell with it. I struck on my mouth and chin. I wear false teeth, and the blow knocked off a part of them, and forced them into my throat; there they stuck. I did not know anything when you came—being stunned—and could give no account of myself. I gradually came to, and at once found out what was the matter. I thrust my fingers into my throat and turned up and out my teeth, and soon breathed as well as ever." My good-natured neighbor laughed heartily at our trouble, and at our not finding out what was the matter.

*Dr. Samuel Parkman.

There can be no doubt that Mr.—— was in some danger, and none that if the teeth had been forced farther into the fauces he would have died of suffocation before assistance could reach him. His entire unconsciousness, together with the heavy stertor; his swollen, turgid and suffused countenance, looked like apoplexy, produced by the shock, or by some grave lesion of the skull or brain, or both.

Why have dentists and false teeth so rapidly increased of late? You may say that the first demanded the last. But why the first? I am on the shady side of seventy, and have lost but five of my old stock, and they have kindly taken their leave, "making no sign." And how have they been preserved? By daily care through many, many years. A stiff brush, wetted and rubbed on a cake of castile soap, and thorough brushing; then rinsing, or better, washing the mouth after every meal. Very few know how to wash the mouth. Unless the water is directed by the will, the muscles of that office being called into voluntary action, and the water be directed to every part of the mouth, nay, to every tooth, and it can be done, your mouth is not washed, or your teeth cleaned. As I for the most part, year in and year out, take my meals at home—never entertaining, and of course being never entertained—I have abundant opportunity to wash and preserve my teeth. I never had but one tooth filled. This filling did no sort of good, and I do not think I shall have the operation repeated. False teeth can be kept clean. Without great care, however, the breath can never be sweet. For my single self, I would much rather swallow than wear them. You can always tell if they are worn, if the careless wearer be on your windward side. They tell us a Parisian knows the quality and source of all the odors of Paris. The false teeth always tell their story. It seems to me that some rule might be established touching these articles of mouth furniture. They might be used when wanted, as is the pocket hankerchief. For instance, when a man is about to make a speech, or a lady to go to a ball, or when either is about sitting down to dinner, or any other meal, at church for responses, or for singing, &c. &c. At all other times they should be out of the mouth, and in a case or bag (a very small one will answer,) and thus all the accidents of breath, of sleeping with the teeth in, or wearing them when at work, or at boxing, or what not, may be prevented, and all the benefit of false teeth be certainly reached. To show you how careful some of the class referred to are of these adventitious substitutes, I will close with a story.

Miss ——, aged about 47 (?,) was under treatment, when it occurred to me that some acid remedy might be useful, and I prescribed one. Said Miss ——, "will it not hurt my teeth?" I said no, if she would follow some simple directions which I gave her. "I ask," said Miss ——, "because I highly value my teeth, as they cost me quite a sum of money!" I assured her that she might be entirely easy on that score, and might use the acid draught as freely, so far as the teeth were concerned, as if it were mere water.—*Boston Med. and Sur. Jour.* W. C.

